# Novel variants identified in five Chinese families with Joubert Syndrome: a case report

**DOI:** 10.1186/s12920-023-01669-7

**Published:** 2023-09-21

**Authors:** Liwei Fang, Lulu Wang, Li Yang, Xiaoyan Xu, Shanai Pei, De Wu

**Affiliations:** Pediatric Neurorehabilitation Center, Pediatric Department, The First Affiliated Hospital of Anhui Medical University, Hefei, 230000 China

**Keywords:** Joubert syndrome, TCTN2, CPLANE1, INPP5E, NPHP1, CC2D2A

## Abstract

**Background:**

Joubert syndrome (JS) is a group of rare ciliopathies, mainly characterized by cerebellar dysplasia representing the “molar tooth sign (MTS)” on neuroimaging, hypotonia, and developmental delay. Having a complicated genotype-phenotype correlation due to its rich genetic heterogeneity, JS is usually combined with other organic defects affecting the retina, kidney, and liver. This report aimed to present new cases and novel variants of JS.

**Case presentation:**

Five unrelated patients who were diagnosed with JS, with or without typical clinical characteristics, received integrated examinations, including whole-exome sequencing (WES) and Sanger sequencing. We identified nine pathogenic variants in the *TCTN2*, *CPLANE1*, *INPP5E*, *NPHP1*, and *CC2D2A* genes.

**Conclusion:**

Four novel pathogenic mutations in the *TCTN2*, *CPLANE1*, and *INPP5E* genes were reported. The findings broadened the genotypic spectrum of JS and contributed to a better understanding of genotype-phenotype correlation.

**Supplementary Information:**

The online version contains supplementary material available at 10.1186/s12920-023-01669-7.

## Background

Joubert syndrome (JS) is a rare developmental malformation, also known as a ciliopathy, which is mainly inherited in an autosomal recessive manner [1]. According to few epidemiological investigations, the prevalence of JS was roughly estimated to be between 1 and 80,000 and 1 in 100,000 [2, 3]. Because of the tremendous genetic heterogeneity of JS, multiple cilium-related genes have recently been discovered as causative in JS by booming genetic testing techniques. First described in 2004, mutations in *NPHP1* and *AHI1* associated with JS came to light [4, 5]. So far, over 40 genes have been linked to JS, and the related pathogenic variants account for more than 60% of the identified variants in different JS cohorts [6]. JS-causative genes encode proteins participating in the formation and function of the basal body and transition zone (TZ) of primary cilium [7], which not only function as cell sensors in almost all types of cells but also control several developmental signaling pathways, including Sonic Hedgehog (Shh), Wnt, and planer cell polarity [2]. The aberrant primary cilia caused by pathogenic variants in these genes lead to abnormal embryonic development. Aside from the commonly observed autosomal recessive pattern of inheritance, an X-linked recessive manner is particularly associated with pathogenic variants in the *OFD1* gene, and recently an autosomal dominant pattern is unveiled by causative variants in the *SUFU* gene [8, 9].

The classical features of JS include the “molar tooth sign (MTS)” on neuroimaging, hypotonia with subsequent ataxia, and developmental delay (DD) or intellectual disability [10]. The canonical MTS characterized by cerebellar abnormalities portraying cerebellar vermis hypoplasia, thickened, elongated, and horizontally oriented superior cerebellar peduncles, and fourth ventricle enlargement [11]. Clinical evidence of MTS is necessary for the diagnosis of JS [12] and it could be mild in some patients found with thinner cerebellar peduncles and slight vermis dysplasia [5, 13]. In addition to the characteristics mentioned, abnormal respiratory patterns, such as alternating tachypnea or apnea after delivery, and abnormal eye movements, including nystagmus, strabismus, and oculomotor apraxia, are frequently observed in patients [14]. Furthermore, depending on the severity of the disease, a high proportion of individuals with JS had concomitant organ abnormalities, which included retinal involvement, renal disease, and hepatic fibrosis [15]. The varying organ abnormalities in JS patients could be explained by JS-causative gene deficiencies in corresponding type of cells, including kidney epithelial cells, retinal photoreceptors, and nerve cells [16, 17].

Previously, a group of diseases with MTS and typical clinical features of JS combined with systemic involvements were named “JS-related disorders”. Such diseases were divided, based on associated comorbidities, into clinical subtypes as follows: classic or pure JS, JS with retinal disease (JS-Ret), JS with renal disease (JS-Ren), JS with oculorenal disease (JS-OR), JS with hepatic disease (JS-H), JS with oral-facial-digital features (JS-OFD), JS with acro-callosal features (JS-AC), and JS with Jeune asphyxiating thoracic dystrophy (JS-JATD). “Joubert syndrome” is now used as a general term for this group of diseases [2].

In this study, we described five patients with or without the typical clinical symptoms of JS, and using whole-exome sequencing (WES), we pointed out four novel pathogenic mutations in the *TCTN2*, *CPLANE1*, and *INPP5E* genes that enriched the genotypic spectrum of JS.

## Case presentation

### Clinical manifestations

Patient 1 was a 3-month-old boy admitted to our department due to hypotonia. The patient, who was the third child in the family, was delivered vaginally at term and weighed 3280 g at birth. His parents insisted on the delivery, despite of the fetal brain MRI performed at 28 weeks of gestation suggesting the cerebellar vermis was absent. The patient also had a hearing impairment and failed to control his head with a soft neck. Gesell scores for the patient indicated a moderate to severe DD: 40 in gross motor, 54 in fine motor, 40 in adaptability, 40 in language ability, and 34 in social ability. His cranial MRI records revealed a typical MTS, an enlarged fourth ventricle, and cerebellar vermis dysplasia. However, the images could not be recovered due to a data disaster. Other routine physical exams and biochemical tests were all normal. His parents and the 6-year-old sister were healthy. The 12-year-old sister, showing claw-like hands with slender, curving fingers and intellectual disability, was unable to walk until she was five years old and fell frequently. We failed to follow up on this patient and his family members due to the COVID-19 outbreak in 2019.

**Fig. 1:**
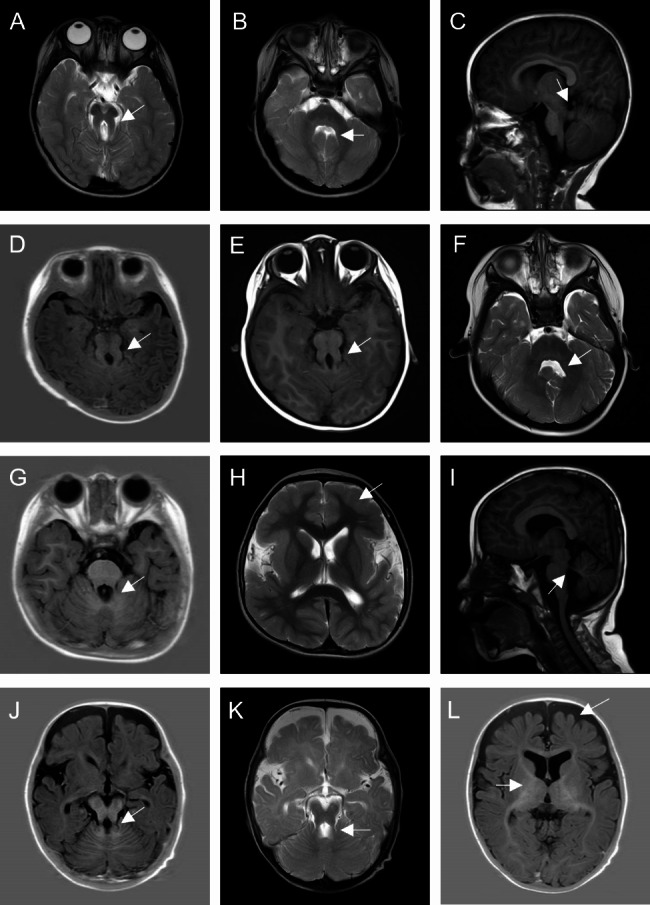
MRI results of four patients. (A-C) Patient 2.(A) Axial T2-weighted image shows cerebellar vermis hypoplasia, a deepened interpeduncular fossa and thickened, elongated superior cerebellar peduncles (arrow), presents a “molar tooth sign”; (B) Enlargement of the fourth ventricle, “bat-wing”(arrow); (C) Parasagittal T1-weighted image showed thickened, elongated, and horizontally oriented superior cerebellar peduncles (arrow); (D-F) Patient 3. (D) Axial T1-weighted image (7 months) shows a blurry deepened interpeduncular fossa and thickened, elongated superior cerebellar peduncles (arrow); (E) Axial T1-weighted image (3 years) shows vermian hypoplasia, thickened, elongated superior cerebellar peduncles (arrow); (F) Axial T2-weighted image (3 years) shows a “bat-wing” of the fourth ventricle; (G-I) Patient 4. (G) Axial T1-weighted image shows slight vermian dysplasia and thin but elongated cerebellar peduncles (arrow); (H) Axial T2-weighted image shows thickened cortex, sparse gyri, and shallow sulci especially in the bilateral frontotemporal lobes (arrow); (I) Parasagittal T1-weighted image shows cerebellar hypoplasia, thin, elongated and horizontally oriented cerebellar peduncles (arrow); (J-L) Patient 4. (J-K) Axial T1 and T2-weighted image shows thin and elongated cerebellar peduncles (arrow); (L) The subarachnoid space is widened bilaterally in the frontotemporal regions (arrow), the ventricular system is enlarged, and the volume of white matter is less than peers, suggesting white matter dysplasia

Patient 2, female, the only child, was admitted to our department at 9 months due to delayed developmental milestones. She had hypotonia, sitting unsteadily and making incomprehensible sounds. Her mother was treated with levothyroxine because of hypothyroidism during pregnancy, though, the patient was born at term with a birth weight of 3000 g and with a normal thyroid function. However, she had poor visual fixation and esotropia after birth. At the age of 10 months, her Gesell assessment revealed a moderate to severe DD: 35 in gross motor, 35 in fine motor, 35 in adaptability, 40 in language ability, and 40 in social ability. Cranial MRI showed a typical MTS and “batwing” appearance of fourth ventricle (Fig. 1A-C). No abnormalities were detected in routine physical exams and biochemical tests. The phenotypes of her parents were normal and there was no relevant family history. The patient has been receiving regular rehabilitation therapies in our department and her Gesell scores at the age of two has improved to 37, 57, 55, 57, and 51, indicating mild to moderate DD. By the time she was three years old, she had a better visual fixation and chasing ability, despite the limited abduction of her left eye. She was also able to understand straightforward instructions, talk in simple words, laugh, and develop fair hand functions. However, hypotonia remained and she could not stand or walk alone.

Patient 3, male, was admitted to our department due to abnormal ocular movements at 7 months. He suffered from downward-limited eye movement, horizontal nystagmus, binocular exotropia, and head tremors. The patient was the second child of the family and born at term with a birth weight of 3100 g. On the ninth day after birth, he developed pathological jaundice, which was improved after exchange transfusion and phototherapy. At 7 months, a Gesell assessment was performed due to delayed motor and linguistic milestones, including neck weakness, wobbly sitting, and incomprehensible pronunciation. The results suggested mild DD: 46 in gross motor, 65 in fine motor, 46 in adaptability, 78 in language capacity, and 65 in social ability. His brainstem auditory evoked potential test indicated moderate hearing impairment. Cranial MRI revealed MTS, which was characterized by the deepening of interpeduncular fossa and the thickening and lengthening of superior cerebellar peduncles (Fig. 1D-F). Regular physical examinations and biochemical tests were normal. The patient’s sister likewise suffered from hypotonia, strabismus, severe myopia, and nystagmus. Follow-up showed that even though the patient turned 3 years old, he could only speak, crawl, and stand with assistance while the ocular abnormalities still remained.

Patient 4 was a 5-year-old boy admitted to our department due to delayed motor and language milestones. He was unable to stand and walk alone and he had poor hand functions and unconscious vocalization. The patient was one of the two living births in a family that had 5 unexplained miscarriages and his older brother was healthy. He was delivered via cesarean section at full term, weighing 2800 g. At the age of 26 months, his Gesell scores showed a severe DD: 48 in gross motor, 33 in fine motor, 31 in adaptability, 33 in language ability, and 38 in social ability. He had seizures at 28 months and the seizure symptoms disappeared after taking sodium valproate. Besides, broad forehead, slightly depressed nasal bridge, low-set ears, micrognathia, adduction deformity of thumb, and slender toes were noticed, along with hypertonia and limited function of the right limbs. Now his EEG result was normal, but blood tests showed a decreased level of red cells, hemoglobin, hyperuricemia, and increased level of alkaline phosphatase. Cranial MRI revealed thickened cortex, sparse gyri, shallow sulci especially in the bilateral frontotemporal lobes, thin but elongated cerebellar peduncles and slight vermis dysplasia (Fig. 1G-I). The follow-up to the age of 7 showed that his DD and hypertonia were not improved.

Patient 5, male, the family’s first child, was admitted to our department due to delayed developmental milestones. He was delivered vaginally at term and weighed 4090 g at birth. Nystagmus and impaired eye movement were discovered during a physical examination. At 7 months, he was unable to sit alone and make comprehensible sounds. Gesell scores showed mild DD: 70 in gross motor, 80 in fine motor, 80 in adaptability, 70 in language ability, and 80 in social ability. Cranial MRI exhibited MTS with a deepened interpeduncular fossa, lengthened superior cerebellar peduncles, white matter abnormalities, and enlarged ventricular system (Fig. 1J-L). Abdominal ultrasonography showed he had an enlarged liver that the lower margin of the liver extended 29 mm beyond the lower margin of the right costal arch. Other physical examinations and biochemical tests were normal. The patient’s uncle had a history of seizures. A follow-up examination revealed that the patient could play and sit independently at 11 months old; however, his motor and linguistic skills fell behind those of his peers, and he still had horizontal nystagmus.

The details of the clinical characteristics of all five patients were provided in Table 1.

**Table 1:** Clinical features of the five patients diagnosed with Joubert syndrome

Features	Patient 1 (TCTN2-JS)	Patient 2 (CPLANE1-JS)	Patient 3 (INPP5E-JS)	Patient 4 (NPHP1-JS)	Patient 5 (CC2D2A-JS)
Age at diagnosis	3 months	9 months	7 months	5 years	7 months
Sex	Male	Female	Male	Male	Male
MTS	+	+	+	mild	mild
Dystonia	Hypotonia	Hypotonia	Hypotonia	Hypertonia	Hypertonia
Developmental delay	+	+	+	+	+
Ocular Manifestation		Strabismus	Strabismus/ nystagmus		Nystagmus
Renal involvement					
Liver involvement					
Seizure				+	
Craniofacial dysmorphisms				+	
Postaxial polydactyly					
Others	Hearing impairment		Hearing impairment		

### Genetic tests

**Fig. 2:**
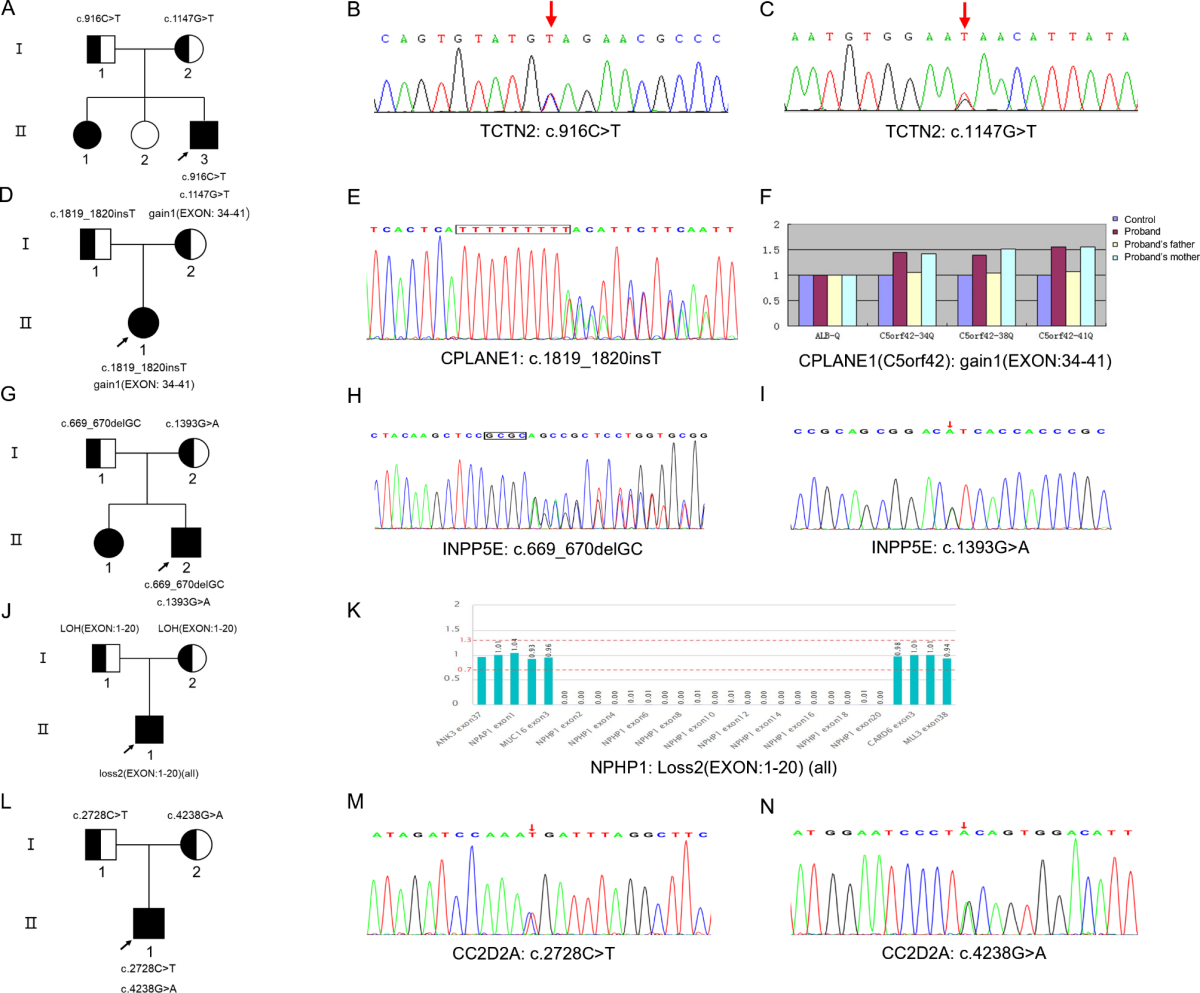
Pedigree diagrams and genetic variants of the patients. (A-C) patient 1. (A) The pedigree diagram of the family, the proband’s parents and the younger sister were phenotypically normal; the proband’s elder sister carried the same variation with the proband. (B) Sanger sequencing confirmed heterozygous c.916 C > T and (C) heterozygous c.1147G > T in the *TCTN2* gene; (D-F) patient 2.(D) The pedigree diagram; (E) Sanger sequencing confirmed a heterozygous c.1819_1820insT variant in the *CPLANE1* gene; (F) Fluorogenic quantitative PCR result for heterozygous *CPLANE1* exon 34–41 duplication, the ALB gene was used as the reference gene; (G-I) patient 3.(G) The pedigree diagram of the family, the proband’s sister carried the same variation with the proband; (H) Sanger sequencing result shows a heterozygous c.669_670delGC variant in the *INPP5E* gene; (I) Sanger sequencing confirmed a heterozygous c.1393G > A variant in the family; (J,K) patient 4.(J) The pedigree diagram; (K) Schematic representation of capture efficiency for homozygous *NPHP1* exons 1–20 deletion; (L-N) patient 5.(L) The pedigree diagram; (M) Sanger sequencing confirmed a heterozygous c.2728 C > T variant and (N) a heterozygous c.4238G > A variant in the *CC2D2A* gene

After obtaining prior written consent of the parents, venous blood samples from all patients and their parents were collected and sent to the Chigene (Beijing) Translational Medical Research Center Co. Ltd. (Beijing, China) for trio-based WES. Sanger sequencing and the American College of Medical Genetics and Genomics (ACMG) clinical practice guidelines [18] were applied for variants interpretation in this study. To be specific, Patient 1 carried a compound heterozygous variation in the *TCTN2* gene consisting of 2 variants, c.916 C > T (p.Q306*) and c.1147G > T (p.E383*) (NM_024809.5) (Fig. 2A-C); Patient 2 had two variants in the *CPLANE1* gene, one is c.1819_1820insT(p.Y607Lfs*12) (NM_023073.4), the other is a haploid replication of exon 34–41 (NM_023073.4), detected by fluorogenic quantitative PCR (Fig. 2D-F); Patient 3 carried a complex heterozygous variation in the *INPP5E* gene consisting of 2 variants, c.669_670delGC (p.A223Afs*66) and c.1393G > A (p.V465I) (NM_019892.6) (Fig. 2G-I); Patient 4 had a homozygous deletion in the *NPHP1* gene (exon 1–20) (NM_000272.5), while both his parents sheltered a heterozygous deletion in the *NPHP1* gene (exon 1–20) respectively (Fig. 2J-K); Patient 5 carried a compound heterozygous variation in the *CC2D2A* gene composed of 2 variants, c.2728 C > T (p.R910*) and c.4238G > A (p.C1413Y) (NM_001080522.2) (Fig. 2L-N). Overall, four novel variants, *TCTN2* Q306* and E383*, *CPLANE1* Y607Lfs*12, *INPP5E* A223Afs* were identified. (Table 2)

**Table 2:** Gene variants identified in this study

Patient No.	Variant No.	Gene (transcript)	Nucleotide change	Amino acid change	Variant type	ACMG* level (evidence)
1	1	*TCTN2* (NM_024809.5)	c.916 C > T	p.Q306*	Nonsense	Pathogenic (PVS1 + PM2-Supporting + PM3)
	2		c.1147G > T	p.E383*	Nonsense	Likely pathogenic (PVS1 + PM2-Supporting)
2	1	*CPLANE1* (NM_023073.4)	c.1819_1820insT	p.Y607Lfs*12	Frameshift	Pathogenic (PVS1 + PM2 + PP5)
	2		dup exon(34–41)	uncertain	CNV*	Likely pathogenic (PVS1-S + PM2)
3	1	*INPP5E* (NM_019892.6)	c.669_670delGC	p.A223Afs*66	Frameshift	Likely pathogenic (PVS1 + PM2)
	2		c.1393G > A	p.V465I	Missense	Likely pathogenic (PM1 + PM2 + PM3 + PP3)
4	1	*NPHP1* (NM_000272.5)	del exon(1–20)	null	CNV*	Pathogenic (PVS1 + PM2 + PM3-Supporting)
5	1	*CC2D2A* (NM_001080522.2)	c.2728 C > T	p.R910*,711	Truncation	Likely pathogenic (PVS1 + PM2)
	2		c.4238G > A	p.C1413Y	Missense	Uncertain significance (PM3 + PP3)

Note: *ACMG: The American College of Medical Genetics and Genomics; *CNV: copy number variant

## Discussion

The diagnosis of JS takes MTS as a necessary criterion, classically characterized by thickened and elongated superior cerebellar peduncles, with the major manifestations including hypotonia and DD. As one of the ciliopathies, JS also has overlapping symptoms with Meckel syndrome, nephronophthisis or other diseases, especially the extra-neuro symptoms such as polydactyly, retinal dystrophy, polycystic kidney, and renal/liver fibrosis [7]. In this study, all five patients showed cerebellar hypoplasia with MTS and varying degrees of DD. After excluding other malformations of cerebellar development based on neuroimaging, JS was initially diagnosed. However, milder forms of MTS were discovered in patients 4 and 5, along with dysplasia of the cortex or white matter. They also suffered from hypertonia, rather than hypotonia in typical JS, and Patient 4 in addition had seizures. It raises a possibility that white matter dysplasia or seizures triggered hypertonia in these patients. At the same time, three patients manifested ocular abnormalities (Patient 2, 3, 5), which are common symptoms of JS. Besides, we did not find any other organ defects. To further build the connections between phenotype and genotype, we identified five variations in the *TCTN2*, *CPLANE1*, *INPP5E*, *NPHP1*, and *CC2D2A* genes, confirming the JS diagnosis.

TCTN2 localizes to the outer compartments of TZ [19] and is crucial in the regulation of ciliogenesis and embryonic development, especially the development of the nervous system [20]. Shown by genetic tests, Patient 1 had a compound heterozygous variation in the *TCTN2* gene comprising of previously unreported mutations c.916 C > T (p.Q306*) and c.1147G > T (p.E383*). Compared to other JS-associated genes, *TCTN2* gene mutations are more likely to result in intellectual disability and less likely to result in renal, hepatic, or retinal involvement [21]. Similarly, Patient 1 had a severe DD with no other organ defects.

A component of TZ, CPLANE1 is commonly associated with JS cases, of which over 125 mutations were identified, accounting for about 8–14% of all JS patients [22, 23]. These mutations in *CPLANE1* lead to dysfunction of TZ, abnormalities of ciliogenesis and Shh signal transduction, causing defects in cerebellar developmental [23]. From Patient 2, the haploid replication of exons 34–41 in the *CPLANE1* gene is the latest discovery. The other genomic alteration c.1819_1820insT in the *CPLANE1* gene is novel, but the amino acid change p.Y607Lfs*12 (rs777686211) shares the same result with a previously reported case (c.1819delT, p.Y607Lfs*6) [22].

NPHP1 and CC2D2A similarly function as essential components of TZ, playing roles in ciliary development. In terms of Patient 4, the homozygous deletion in the *NPHP1* gene (exon 1–20) results in loss of function of the protein, which has been observed in several other JS patients [24, 25]. Notably, as the unusual MRI finding of Patient 4, NPHP1-associated JS cases have repeatedly been reported to exhibit milder “MTS” [5, 13, 25], suggesting that NPHP1 defects may have less impact on cerebellar development. Nonetheless, seizures have not been reported in NPHP1-associated JS, while such symptom have been linked to other JS-related genes, such as the *CC2D2A* and *AHI1*. The relationship between JS genotype and seizure is ambiguous, but seizure management is similar for JS and non-JS individuals [26]. As for Patient 5, c.4238G > A (p.C1413Y) in the *CC2D2A* gene was previously reported in JS [25] and nephronophthisis [27], and c.2728 C > T (p.R910*) was also found in a case of prenatally diagnosed JS [28].

Unlike the previously mentioned proteins, INPP5E localizes to various subcompartments of primary cilium in a TZ-dependent manner and regulates ciliogenesis through phosphoinositide 3-kinase signaling pathway [29]. The variant c.1393G > A (p.V465I) in the *INPP5E* gene was previously detected in a patient with inherited retinal degenerations [30], and c.669_670delGC (p.A223Afs*66) is firstly found in JS.

Since there is no curative therapy for patients with JS, only regular follow-up and symptomatic treatment are practicable. Although a gene-targeted therapy for JS has entered the early stage of research, the extreme genetic heterogeneity of JS and the differences between human and animal models make the research exceedingly difficult [31]. Prenatal screening accordingly becomes crucial, especially for families having a JS-related history. Routine prenatal ultrasound and fetal brain MRI can be used for early screening for cerebellar malformations, but they may not work well due to the inability to detect a typical MTS, which needs genetic tests to confirm the prenatal diagnosis [8, 32, 33]. Careful follow-up and rehabilitation training may enhance the patients’ daily activities for individuals who receive their diagnosis in the early stage of life. Regarding possible involvement in several organs, such as retinal atrophy and renal/liver disorders, it is necessary to monitor the renal status and renal/liver function of the patients for a long period [31]. To date, no patient in this study has shown extra organic involvement. All patients received individual, scientific, and regular rehabilitation, including transcranial magnetic/ultrasound stimulation, physical therapy, occupational therapy, speech therapy and traditional Chinese medicine treatment, such as acupuncture, moxibustion and massage. Follow-ups showed that Patient 2 has made considerable progress, while Patient 4 and 5 improved slowly due to a complex encephalodysplasia and a history of seizures.

Indeed, our research had certain limitations. First, we cannot regain the MRI images of Patient 1 and lost the follow-ups, which makes it an imperfect case. Second, patients from other cities cannot receive timely rehabilitation and developmental assessments because of the COVID-19 epidemic. Third, the siblings of Patient 1 and 3 also had related symptoms but they did not receive proper diagnosis and treatment due to family reasons.

In conclusion, we diagnosed JS in five patients, and the WES data revealed four novel variants in the *TCTN2*, *CPLANE1*, and *INPP5E* genes. Our findings enrich the spectrum of pathogenic variants in JS and provide more practical experience in genetic diagnosis and counseling. Hoping that our reports could shed light on the clinical diagnosis of JS and trigger further studies on therapies.

### Electronic supplementary material

Below is the link to the electronic supplementary material.

Supplementary Material 1

## Data Availability

The majority of data presented in the study are included in the article, further inquiries can be directed to the corresponding author.

## Abbreviations

JS: joubert syndrome
MTS: molar tooth sign
WES: whole-exome sequencing
TCTN2: tectonic-2
CPLANE1: ciliogenesis and planar polarity effector complex subunit 1
INPP5E: inositol polyphosphate-5-phosphatase
NPHP1: nephrocystin 1
CC2D2A: coiled-coil and C2 domain-containing protein 2 A
TZ: transition zone
Shh: sonic hedgehog
DD: developmental delay
MRI: magnetic resonance imaging
EEG: electroencephalogram
ACMG: American College of Medical Genetics and Genomics
PCR: polymerase chain reaction
